# Physical, Thermal, and Optical Properties of Mn^2+^ and Nd^3+^ Containing Barium Phosphate Glasses

**DOI:** 10.1021/acsphyschemau.4c00020

**Published:** 2024-04-26

**Authors:** José A. Jiménez

**Affiliations:** Department of Biochemistry, Chemistry, and Physics, Georgia Southern University, Statesboro, Georgia 30460, United States

**Keywords:** optical properties, phosphate glasses, photoluminescence, physical properties, solar spectral converters, thermal properties

## Abstract

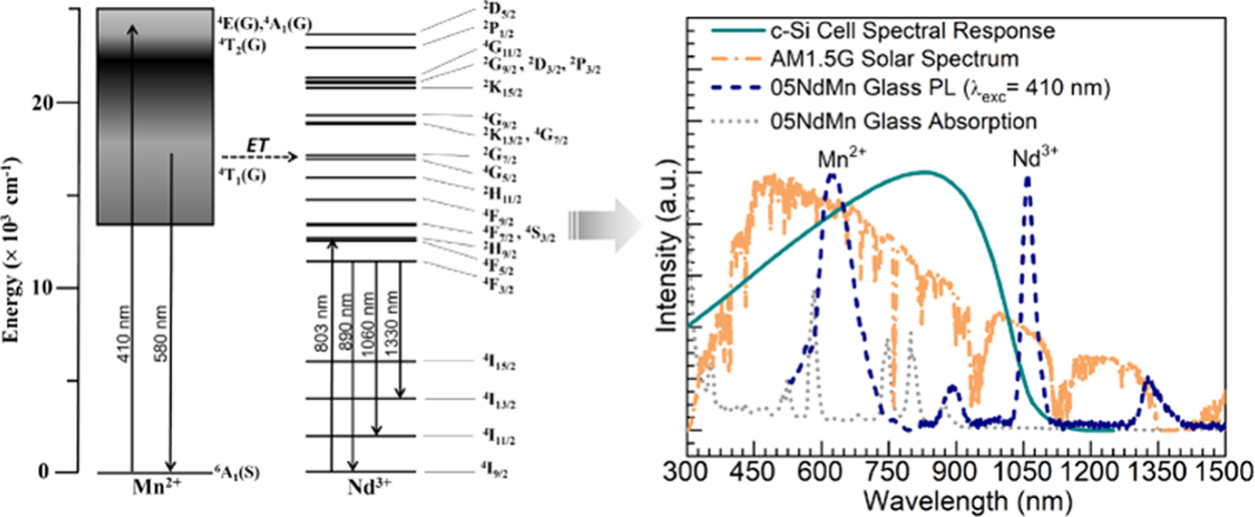

This work reports on various properties and analysis of optical interactions in phosphate glasses containing red-emitting Mn^2+^ and near-infrared (NIR)-emitting Nd^3+^ ions, which are of interest for energy applications and solar spectral converters. The glasses were made by melting with 50P_2_O_5_–(48 – *x*)BaO–2MnO–*x*Nd_2_O_3_ (*x* = 0, 0.5, 1.0, and 2.0 mol %) nominal compositions and characterized by X-ray diffraction, density and related physical properties, differential scanning calorimetry, dilatometry, UV–vis–NIR optical absorption, and photoluminescence spectroscopy with decay kinetics analysis. The glasses were X-ray amorphous, wherein the physical and thermal properties of the Mn^2+^/Nd^3+^-codoped glasses were largely impacted by Nd_2_O_3_ contents. The optical absorption spectra supported the occurrence of Mn^2+^ ions and the lack of Mn^3+^ in the codoped glasses, while the absorption due to Nd^3+^ ions increased steadily with Nd_2_O_3_ contents. Analyzing the glass absorption edges via Tauc and Urbach plots was further pursued for a comparison. The photoluminescence evaluation showed a consistent suppression of the emission from Mn^2+^ ions with increasing Nd^3+^ concentration, while the decay kinetics revealed shorter lifetimes in connection with increased Mn^2+^ → Nd^3+^ transfer efficiencies. Excitation of Mn^2+^ at 410 nm, however, led to the Nd^3+^ NIR emission being most intense for 1.0 mol % Nd_2_O_3_, despite the ^4^F_3/2_ emission decay analysis showing lifetime shortening throughout. Considering the compromise between red and NIR emissions, the Mn-containing glass doped with 0.5 mol % Nd_2_O_3_ is put in perspective with the concept of solar spectral conversion.

## 1 Introduction

Neodymium-doped glasses have been studied extensively, given their near-infrared (NIR) emission useful for high-power lasers^1−11^ and attractive for solar spectral converters.^12−14^ Seeking to boost the emission from the Nd^3+^ (*f*^3^) ions valuable for applications, the codoping of the glasses with different ions intended to act as sensitizers has been proposed, which includes Mn^2+^,^15,16^ Cr^3+^,^17^ Sn^2+^,^18^ Cu^+^,^14,19^ and Ag^+^.^20,21^ Among these, the use of the red-emitting Mn^2+^ ions is particularly interesting, given the potential to be used in conjunction with NIR-emitting rare earth in glasses to be exploited for solar cell applications.^22^

Concerning the different glass hosts available, phosphate-based glasses are specifically desirable for their high metal solubility, appropriate thermo-mechanical properties, and manufacturability.^1−10^ With regards to Mn^2+^/Nd^3+^ codoping, over half a century ago, Shionoya and Nakazawa^15^ and Melamed et al.^16^ reported on the utility of the sensitizing strategy to achieve lasing in phosphate glass hosts. The pioneering work was mostly done on calcium phosphate glasses emphasizing laser action but not scrutinizing additional glass properties.^15^ Further on, Ajithkumar and Unnikrishnan^23^ studied the energy transfer from Mn^2+^ to Nd^3+^ in various sodium phosphate glasses, leading to the conclusion that the dipole–dipole mechanism is responsible for the sensitizing effect. Nevertheless, the authors’ communication did not consist of a comprehensive study of the different physical properties of the glasses.^23^ More recently, Zhang et al.^24^ reported on the photoluminescence (PL) down-shifting and NIR emission from Nd^3+^ centers in 50P_2_O_5_–(50 – *x*)SrO–*x*MnO–*y*Nd_2_O_3_ metaphosphate glasses with *x* = 0, 10, 20, 30, 40, 50, and *y* = 0 and 0.5 mol %. Still, the authors focused merely on evaluating the optical absorption and luminescent properties of the glasses.^24^ Consequently, there seems to be room for investigating further the fundamental physical and optical properties of Mn^2+^/Nd^3+^-codoped phosphate glasses and their potential for energy-related applications.

Alkaline earth metals are often preferred as network modifiers in phosphate glasses^15,24,25^ as these improve chemical durability, which becomes a challenging issue when using alkali metals such as sodium.^23^ Among these, large-radius Ba^2+^ cations are especially useful for achieving adequate physical and optical properties in phosphate-based glass matrices.^1,2,8,14,25^ Nonetheless, thus far, only the alkaline earth metals calcium and strontium have been employed for studying Mn^2+^/Nd^3+^ codoping.^15,24^ Therefore, in the present work, Mn^2+^/Nd^3+^-codoped barium phosphate glasses were prepared with 50P_2_O_5_–(48 – *x*)BaO–2MnO–*x*Nd_2_O_3_ (0 ≤ *x* ≤ 2 mol %) nominal compositions and studied thoroughly with respect to physical, thermal, and optical properties. Following glass synthesis by melt-quenching, a detailed experimental investigation was carried out, incorporating measurements made by X-ray diffraction (XRD), densitometry, differential scanning calorimetry (DSC), dilatometry, UV–vis–NIR spectrophotometry, and PL spectroscopy with emission dynamics assessment. The purpose is to analyze the different physicochemical parameters extracted as a function of the Nd^3+^ concentration and ultimately assess the optical performance of the codoped glasses with a view toward solar spectral conversion.

## 2 Experimental Section

### 2.1 Materials

The melt-quenching technique was used to prepare the glasses with 50P_2_O_5_–(48 – *x*)BaO–2MnO–*x*Nd_2_O_3_ (*x* = 0, 0.5, 1.0, and 2.0 mol %) nominal compositions using as raw materials P_2_O_5_ (Thermo Scientific, 98%), BaCO_3_ (Thermo Scientific, 99.8%), MnCO_3_ (Alfa Aesar, 99.9%) and Nd_2_O_3_ (Thermo Scientific, 99.99%). The different compounds were weighed in the appropriate quantities (about 25 g batches), targeting the molar compositions summarized in Table 1, which also shows the assigned glass codes. The reagents were thoroughly mixed, melted at 1150 °C for 15 min in porcelain crucibles under ambient atmosphere, and quenched in heated steel molds. The amount of MnCO_3_ in the Mn-containing glasses was kept fixed at 2.0 mol %, which was added in substitution of BaCO_3_. The decomposition of such carbonates at high temperatures is expected to take place similarly as
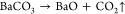
1
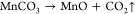
2However, for manganese(II), there is a susceptibility for oxidation, given that the melting was carried out in the air atmosphere. The amount of MnCO_3_ was then chosen at 2.0 mol %, seeking a considerable effect on glass properties while avoiding the significant occurrence of Mn^3+^ (not detected optically, *vide infra*), which could arise from Mn^2+^ oxidation in the melts. The addition of 2.0 mol % MnCO_3_ together with increasing quantities of Nd_2_O_3_ added as 0.5, 1.0, and 2.0 mol % replacing BaCO_3_ was then intended to evaluate the impact of the Nd_2_O_3_ codoping on the various properties assessed. To aid in the evaluation, the barium phosphate (BP) glass host and a glass containing merely 2.0 mol % Nd_2_O_3_ (labeled 2Nd) were made for reference purposes.

**Table 1:** Glass Codes and Nominal Molar Concentrations of the Reagents Employed to Prepare the Glasses

Glass	P_2_O_5_ (mol %)	BaCO_3_ (mol %)	MnCO_3_ (mol %)	Nd_2_O_3_ (mol %)
BP	50.0	50.0		
2Mn	50.0	48.0	2.0	
05NdMn	50.0	47.5	2.0	0.5
1NdMn	50.0	47.0	2.0	1.0
2NdMn	50.0	46.0	2.0	2.0
2Nd	50.0	48.0		2.0

**Figure 1:**
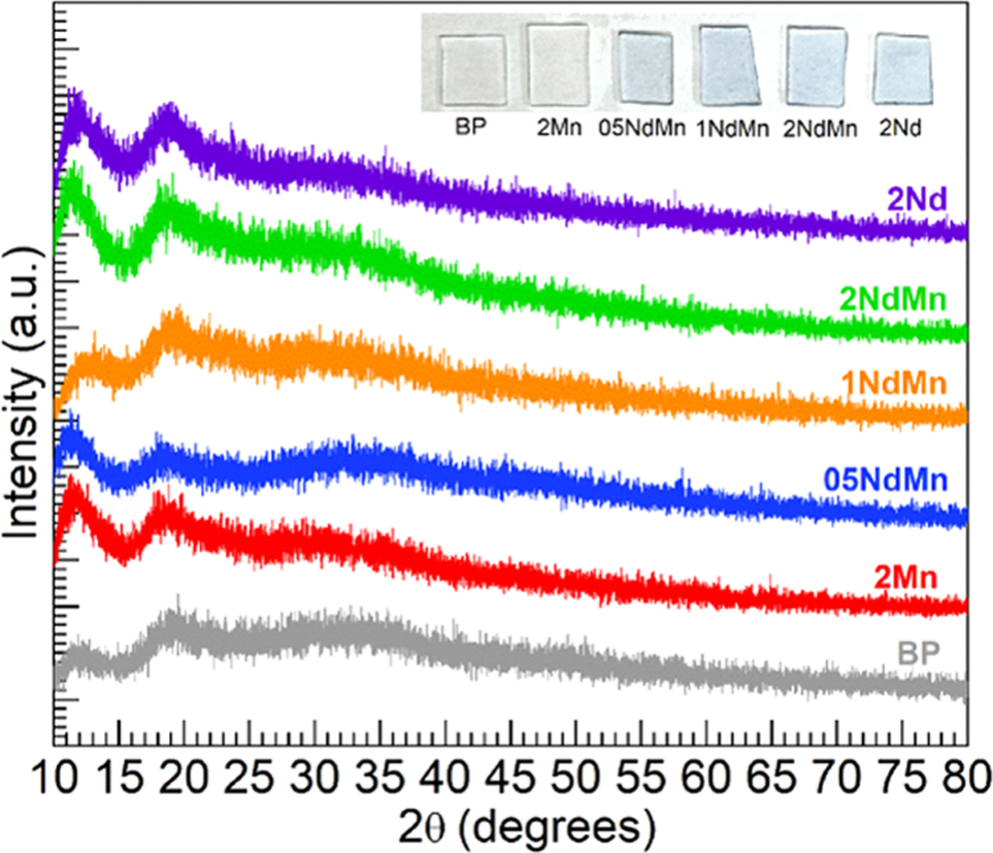
XRD patterns obtained for the various glasses within the 10° ≤ 2θ ≤ 80° range using Mo-*K*_α_ radiation. The inset contains a photograph of slabs for the different glasses.

Immediately after the quenching of the glass melts, the glasses were annealed at 420 °C (below the glass transition temperature, *vide infra*) for 3 h to remove stress. After cooling to room temperature (RT), the glasses were then cut and polished to ∼1 mm thick slabs for optical measurements. The BP and 2Mn glasses appeared clear and colorless to the naked eye, whereas the Nd-containing glasses exhibited a purple hue, which intensified with Nd_2_O_3_ content (a photograph of samples for the different glasses is shown in the inset of Figure 1). Glass pieces were also crushed by mortar and pestled for XRD and DSC. Glasses were also quenched separately as glass rods and cut to a length (*L*) of about 2.54 cm for dilatometric measurements.

### 2.2 Measurements

XRD was performed to verify the noncrystalline nature of the glasses as powders (crushed by mortar and pestle) with a PANalytical Empyrean X-ray diffractometer operating at RT using Mo-*K*_α_ radiation (λ = 0.71 Å). The acceleration voltage and current used were 60 kV and 40 mA, respectively.

Glass densities were measured by the Archimedes principle in a Mettler-Toledo XSR Analytical Balance with distilled water as immersion liquid. The determinations were done at RT in triplicate, and the averages were reported (uncertainties in the third decimal place). Other physical parameters deemed useful for characterizing the glasses were also calculated in accordance with corresponding formulas.^26,27^ The average molar mass (*M*_av_) was calculated by
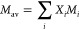
3where *X*_*i*_ and *M*_*i*_ are the mole fraction and molar mass of the *i*^th^ component, respectively. From the measured densities (ρ), the molar volumes (*V*_m_) were obtained as
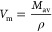
4The concentration of specific ions (*N*_*i*_) in the glasses was calculated with the corresponding mole fractions (*X*_*i*_), the glass densities, and the average molar masses according to
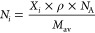
5where *N*_A_ is Avogadro’s constant. The mean interionic distances between like ions (*d*_*i-i*_) were then calculated from the following relation
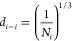
6Finally, the mean distance between different ions (*d*_*i-j*_) where applicable was estimated from
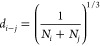
7where *N*_*i*_ and *N*_*j*_ are the corresponding ionic concentrations.

DSC was carried out for sample grains in an SDT650 calorimeter (TA Instruments) in alumina pans using a heating rate of 10 °C/min and a nitrogen gas atmosphere (flow rate at 100 mL/min). The different thermal parameters [glass transition temperature (*T*_g_), onset of crystallization (*T*_x_), and peak crystallization temperature (*T*_c_)] were estimated using the instrument’s software (midpoint-inflection point for *T*_g_). Dilatometry measurements were carried out on the glass rods in an Orton dilatometer (Model 1410B) at a heating rate of 3 °C/min. The determination of the coefficient of thermal expansion (CTE) and the softening temperature (*T*_s_) was then made with the instrument’s software.

UV–vis–NIR optical absorption measurements were performed at RT on the ∼1 mm thick glass slabs with an Agilent Cary 5000 double-beam spectrophotometer. PL spectra were recorded at RT under steady-state conditions with a Horiba Fluorolog-QM spectrofluorometer equipped with an Xe lamp and an InGaAs detector used for the NIR measurements. A Xe flash lamp (∼2 μs pulse duration) was used for collecting emission decay curves.

## 3 Results and Discussion

### 3.1 XRD, Density, and Basic Physical Properties

Figure 1 shows the powder XRD patterns obtained for the different glasses (the photograph in the inset shows samples of the different glasses as slabs). The diffractograms show humps toward small 2θ values, which characterize the long-range structural disorder.^27^ Despite some intensity fluctuations seen with the broad features, the diffractograms do not show discrete crystallization peaks, thus supporting the noncrystalline nature of the glasses.

The average densities obtained for the different glasses are presented in Table 2, together with the physical quantities calculated through eqs 3–7. The density of the 2Mn glass at 3.649 g/cm^3^ is noticed to be somewhat lower than that for the BP host of 3.700 g/cm^3^. This harmonizes with the fact that 2 mol % BaCO_3_ was replaced as raw material in the 2Mn glass with an equal amount of MnCO_3_ with a lower molar mass. Thereafter, keeping the content of MnCO_3_ fixed at 2 mol % while replacing BaCO_3_ with Nd_2_O_3_ in the 05-2NdMn glasses resulted in increased densities within 3.671–3.692 g/cm^3^. The density of the 2Nd glass containing merely 2 mol % Nd_2_O_3_ was then the highest at 3.717 g/cm^3^. The effect of density rising with Nd_2_O_3_ content is analogous to the observed for singly Nd-doped glasses with 50P_2_O_5_–(50 – *x*)BaO–*x*Nd_2_O_3_ (0 ≤ *x* ≤ 4 mol %) nominal compositions.^27^ It is also in harmony with reports for different glass systems where increasing the Nd_2_O_3_ concentration produced higher densities.^2,5,10,11^

**Table 2:** Densities and Other Parameters Related to the Physical Properties of the Different Glasses

parameter	BP	2Mn	05NdMn	1NdMn	2NdMn	2Nd
density, ρ (g/cm^3^)	3.700	3.649	3.671	3.673	3.692	3.717
average molar mass, *M*_av_ (g/mol)	147.64	145.99	146.90	147.82	149.65	151.30
molar volume, *V*_m_ (cm^3^/mol)	39.90	40.01	40.02	40.24	40.54	40.70
Nd^3+^ concentration, *N*_Nd_ (×10^20^ ions/cm^3^)			1.505	2.993	5.943	5.918
Nd^3+^–Nd^3+^ mean distance, *d*_Nd–Nd_ (Å)			18.80	14.95	11.89	11.91
Mn^2+^ concentration, *N*_Mn_ (×10^20^ ions/cm^3^)		3.010	3.010	2.993	2.971	
Mn^2+^–Mn^2+^ mean distance, *d*_Mn–Mn_ (Å)		14.92	14.92	14.95	14.99	
Mn^2+^–Nd^3+^ mean distance, *d*_Mn–Nd_ (Å)			13.03	11.87	10.39	

As seen in Table 2, the average molar mass for the 2Mn glass decreases relative to the BP host; yet, the values increase steadily in the Nd-containing glasses as expected. For the molar volumes, a general trend of increase within the 39.90–40.70 cm^3^/mol range is, however, observed throughout the whole glass series. This is even the case for the 2Mn glass with a lower average molar mass than the BP host. This is because the 2Mn glass has also a lower density, which appears prevailing in the denominator in eq 4. Thereafter, the concomitant increase in molar mass and density in the 05-2NdMn glasses leads to a general trend of increase in the molar volumes. However, the 2Nd glass exhibits the highest molar volume in agreement with the fact that it has no manganese but has barium instead. The Nd^3+^ concentrations, *N*_Nd_, then vary within the 1.505–5.943 × 10^20^ ions/cm^3^ range with the increase in Nd_2_O_3_ contents in the 05-2NdMn glasses. The resulting Nd^3+^ concentration for the 2Nd of 5.918 × 10^20^ ions/cm^3^ is close to the 2NdMn glass with 5.943 × 10^20^ Nd^3+^ ions/cm^3^. The mean Nd^3+^–Nd^3+^ interionic distances, *d*_Nd–Nd_, then decreased from 18.80 Å in the 05NdMn glass to 11.89 Å in 2NdMn. The 2Nd glass with a mean Nd^3+^–Nd^3+^ interionic distance of 11.91 Å is ultimately comparable to the 2NdMn glass. With respect to manganese, by disregarding oxidation, the maximum Mn^2+^ concentrations, *N*_Mn_, were calculated, which show, in Table 2, similar values for the 2Mn and 05Nd-2NdMn glasses. However, even though the glasses were prepared with the same molar amounts of manganese(II) carbonate, a slight decrease in concentration from 3.010 to 2.971 × 10^20^ ions/cm^3^ resulted from a dilution effect as the molar volumes increased in going from the 2Mn to the 0.5-2NdMn glasses. Consequently, the estimated Mn^2+–^Mn^2+^ mean distances in Table 2, *d*_Mn–Mn_, increased slightly in the 14.92–14.99 Å range. Finally, for the codoped glasses, the Mn^2+–^Nd^3+^ mean distances, *d*_Mn–Nd_, are seen in Table 2 to decrease from 13.03 Å in the 05NdMn glass to 10.39 Å in the 2NdMn glass. This last aspect will become a point of focus when considering the impact of increasing Nd_2_O_3_ content on the luminescent properties of the glasses.

### 3.2 Thermal Properties

#### 3.2.1 DSC

**Figure 2:**
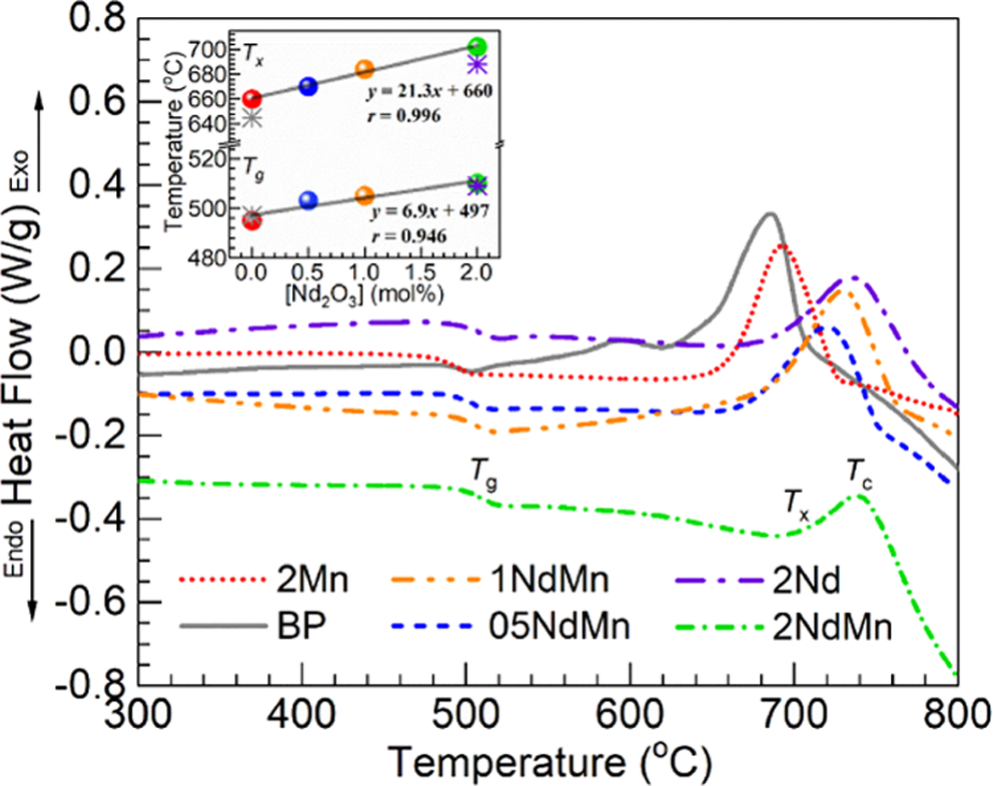
DSC profiles obtained for the different glasses displaying the regions of glass transition temperature (*T*_g_), onset of crystallization (*T*_x_), and crystallization temperature (*T*_c_); regions indicated for the 2NdMn glass (estimated values for all glasses presented in Table 3). The inset is a plot of the *T*_g_ (lower symbols) and *T*_x_ (upper symbols) estimated vs Nd_2_O_3_ concentration in the glasses (spheres—2Mn, 05NdMn, 1NdMn, and 2NdMn; asterisks—BP and 2Nd references); the solid lines are linear fits to the data for the 2Mn, 05NdMn, 1NdMn, and 2NdMn glasses (equations and correlation coefficients, *r*, displayed).

The thermograms obtained from the DSC measurements performed for the various glasses under consideration are shown in Figure 2. From the different thermal profiles obtained covering the 300–800 °C range, the *T*_g_, *T*_x_, and *T*_c_ values were determined for each glass as summarized in Table 3. Also, Table 3 shows the additional parameter, indicating glass stability, Δ*T = T*_x_ – *T*_g_.^28^ The undoped BP glass taken as reference presented a crystallization peak with *T*_c_ around 686 °C and an onset temperature *T*_x_ at 645 °C. The *T*_g_ was estimated at 497 °C, and consequently, the thermal stability factor Δ*T* was 148 °C. The 2Mn glass exhibited similarly a *T*_g_ at 495 °C; however, increased values of *T*_x_ and *T*_c_ were determined at 660 and 693 °C, respectively. An increased glass stability factor was then obtained for the 2Mn glass with Δ*T =* 165 °C. It is also observed in Table 3 that the different thermal parameters tend to increase with Nd_2_O_3_ content in the 05-2NdMn glasses. It turns out that this trend harmonizes with the increase in the 05-2NdMn glass densities in Table 2. The *T*_g_ of the 2Nd reference at 509 °C is, however, comparable to that for the 2NdMn glass at 510 °C, whereas the *T*_x_ and *T*_c_ of the 2Nd glass were lower leading to a smaller Δ*T* of 179 °C.

**Table 3:** Glass Transition Temperature (*T*_g_, Estimated from Midpoint-Inflection Approach), Onset of Crystallization (*T*_x_), Main Peak Crystallization (*T*_c_) Temperature, and Thermal Stability Parameter Δ*T = T*_x_ – *T*_*g*_, Estimated for the Various Glasses from the DSC Profiles (Figure 2)

glass	*T*_g_ (°C)	*T*_x_ (°C)	*T*_c_ (°C)	Δ*T = T*_x_ – *T*_g_ (°C)
BP	497	645	686	148
2Mn	495	660	693	165
05NdMn	503	670	721	167
1NdMn	505	684	730	179
2NdMn	510	702	740	192
2Nd	509	688	736	179

The *T*_g_ values are plotted in the inset of Figure 2 as a function of the Nd_2_O_3_ concentration in the glasses for further evaluation; the spheres are used to represent the data points for the 2Mn, 05NdMn, 1NdMn, and 2NdMn glasses, while the asterisks are used for the BP and 2Nd as references. Regression analysis performed on the *T*_g_ data for the 2Mn and 05-2NdMn glasses yielded a correlation coefficient *r* of 0.946. The obtained intercept of 497 °C was close to the measured *T*_g_ for the 2Mn glass of 495 °C and coincided with the BP glass *T*_g_ (Table 3). Rising *T*_g_ values were similarly seen with Nd_2_O_3_, replacing BaO in the 50P_2_O_5_–(50 – *x*)BaO–*x*Nd_2_O_3_ (0 ≤ *x* ≤ 4 mol %) glass system.^27^ Such outcome is indicative of a glass strengthening effect induced by Nd^3+^ ions and was similarly noticed by Wang et al.,^29^ with increasing Gd^3+^ content in calcium phosphate glasses with 50P_2_O_5_–(50 – *x*)CaO–*x*Gd_2_O_3_ (0 ≤ *x* ≤ 6 mol %) compositions. The *T*_x_ values are similarly plotted together with the *T*_g_ in the inset of Figure 2 as a function of the Nd_2_O_3_ concentration in the glasses. Regression analysis was performed again for the data of the 2Mn and 05-2NdMn glasses, which yielded a correlation coefficient *r* of 0.996, indicating better linearity compared to the *T*_g_. Moreover, the intercept of 660 °C coincides with the *T*_x_ value measured for the 2Mn glass (Table 3). The increasing *T*_x_ behavior characterizing thermal stability improvements also concurs with the reported for the singly Nd-doped barium phosphate glasses^27^ and Gd-loaded calcium phosphate glasses.^29^ The trend for the peak crystallization temperatures as well as the Δ*T = T*_x_ – *T*_g_ thermal stability parameter are also noticed to increase consistently for the 05-2NdMn glasses in Table 3. Thermal property changes such as these have been interpreted in terms of the high ionic field strength of the lanthanide ions substituting other cations with lower field strength.^27,29,30^ Herein, we notice consistency with the report for the singly Nd-doped glasses^27^ concerning this argument. This is because exchanging Ba^2+^ with Nd^3+^ cations in the 05-2NdMn glasses is indicated to lead to a bond-strengthening effect and overall enhanced glass stability with increasing Nd^3+^ concentration. Comparing the results of the 2Mn glass in Table 3 with the 2Nd as a reference also shows that the neodymium has a greater impact than manganese. For comparison, we may consider the ionic radii reported by Shannon^31^ of 0.83 and 0.983 Å for Mn^2+^ and Nd^3+^ cations, respectively, for anticipated coordination numbers of six.^32,33^ The ionic field strengths (*F*) may be calculated with the common formula
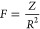
8where *R* is the ionic radius and *Z* is the cation charge.^27^ The resulting *F* values are 2.903 and 3.105 Å^–2^ for Mn^2+^ and Nd^3+^, respectively. This seems rather coherent with the greater impact of neodymium on the thermal properties (Table 3). However, the field strength argument is not satisfactory enough to explain the similar *T*_g_ values for the BP host and the 2Mn glass where Ba^2+^ cations with lower field strength (*F* = 0.992 Å^–2 ^^27^) were being depleted. Regarding the effect of manganese(II), Franco et al.^34^ studied Mn^2+^-doped zinc phosphate glasses of the P_2_O_5_–Na_2_O–ZnO type and reported a slight tendency for the *T*_g_ to increase with MnO content added up to 1 mol %. Recently, Akhrouf et al.^35^ also reported increasing *T*_g_ values for the entire range studied in 25Na_2_O–(30 – *x*)CaO–*x*MnO–45P_2_O_5_ (0 ≤ *x* ≤ 30 mol %) glasses. On the other hand, Pascuta et al.^36^ studied glasses in the *x*MnO–40P_2_O_5_–(60 – *x*)ZnO system with *x* up to 20 mol % and reported a lower *T*_g_ for 5 mol % MnO relative to the undoped glass. Still, the authors mentioned that the *T*_g_ for all samples was around 415 °C and that the parameter was poorly affected by the substitution performed. Herein, we see the resemblance in the fact that similar *T*_g_ values were found for the 2Mn glass and the undoped BP host. Still, a separate study involving different manganese concentrations would be necessary to help clarify this.

#### 3.2.2 Dilatometry

**Figure 3:**
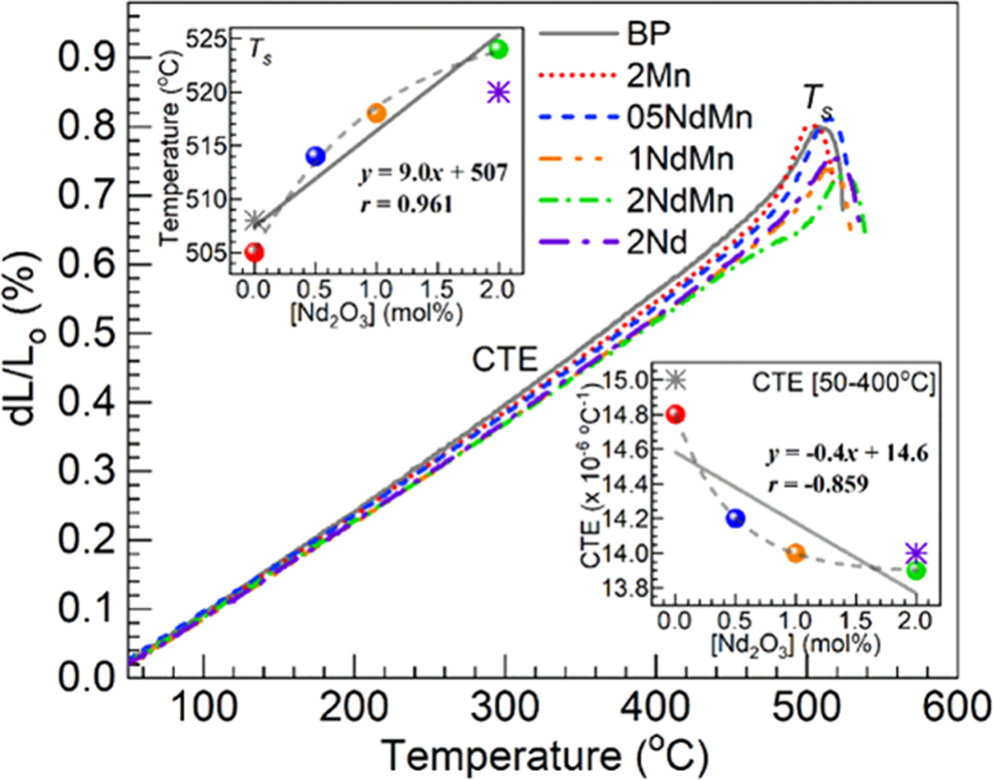
Dilatometric profiles obtained for the different glasses; estimated values of coefficient of thermal expansion (CTE) and softening temperature (*T*_s_) are presented in Table 4. The lower and upper insets are plots of the CTE and *T*_s_ values estimated, respectively, vs Nd_2_O_3_ concentration in the glasses (spheres—2Mn, 05NdMn, 1NdMn, and 2NdMn; asterisks—BP and 2Nd references). The solid lines in the insets are linear fits to the data (equations and correlation coefficients, *r*, displayed); the dashed traces are guides for the eye concerning 2Mn and 05-2NdMn data points.

Complementing the thermal characterization, results from dilatometry measurements are now considered, which are shown in Figure 3. Herein, the evolution of the linear expansion profiles d*L*/*L*_o_ (%) vs temperature was used for the extraction of the parameters of coefficient of thermal expansion, CTE, and dilatometric or softening temperature, *T*_s_.^27^ The CTE values were determined in the 50–400 °C range, whereas *T*_s_ is the peak temperature within the expansion region (considering that the *T*_g_ values were estimated by DSC above, the determination of such by dilatometry was not pursued). The CTE and *T*_s_ values obtained for the different glasses are summarized in Table 4 and are also plotted in the insets of Figure 3 as a function of the Nd_2_O_3_ concentration in the glasses. A general trend to decrease can be observed regarding the CTE in going from the BP host to the 2Mn and 05-2NdMn glasses. Further, the CTE of the 2Nd glass came out at 14.0 × 10^–6^ °C^–1^ coinciding with the 1NdMn glass. The regression analysis performed on the CTE data of the 2Mn and 05-2NdMn glasses as shown in the lower inset of Figure 3 exhibited a rather poor linear correlation with a coefficient *r* = −0.859; the dashed trace was thus included as a guide for the eye concerning the data points involved. Nonetheless, the obtained intercept from the linear fit to the CTE data being 14.6 × 10^–6^ °C^–1^ is close to the CTE measured for the 2Mn glass of 14.8 × 10^–6^ °C^–1^ (Table 4). With respect to the *T*_s_, it is observed in Table 4 that the value of the 2Mn glass at 505 °C is somewhat lower than the estimated for the undoped glass, but thereafter, the *T*_s_ values increase throughout for the 05-2NdMn glasses. Then again, the 2Nd reference has a *T*_s_ lower than the 2NdMn. The tendency for the *T*_s_ to increase with Nd_2_O_3_ content in the 05-2NdMn glasses concurs with the rising trend in the *T*_g_ values obtained from DSC (Table 3), pointing to a glass strengthening effect. Regression analysis performed on the *T*_s_ values for the 2Mn and 05-2NdMn glasses in the top inset of Figure 3 yielded a correlation coefficient *r* of 0.961, higher than the one for the CTE (the dashed trace is also included as a guide for the eye concerning the affected data points). The obtained intercept of 507 °C was also close to the *T*_s_ measured for the 2Mn glass at 505 °C (Table 3). The slope of 9.0 °C/mol % (Figure 3, upper inset) is also not too far from the one obtained for the *T*_g_ of 6.9 °C/mol % (Figure 2, inset).

**Table 4:** Values of Linear Coefficient of Thermal Expansion (CTE, Estimated in the 50-400 °C Range) and Softening Temperature (*T*_s_) Determined for the Different Glasses from Dilatometry (Figure 3)

glass	CTE (×10^–6^ °C^–1^)	*T*_s_ (°C)
BP	15.0	508
2Mn	14.8	505
05NdMn	14.2	514
1NdMn	14.0	518
2NdMn	13.9	524
2Nd	14.0	520

The trends of increasing *T*_s_ and decreasing CTE values observed among the 2Mn and 05-2NdMn glasses generally agree with the field strength arguments.^27,29,30^ For instance, similar trends were seen with Nd_2_O_3_ replacing BaO in the 50P_2_O_5_–(50 – *x*)BaO–*x*Nd_2_O_3_ (0 ≤ *x* ≤ 4 mol %) glass system, suggesting an impact of high-field strength Nd^3+^ ions replacing Ba^2+^ ions.^27^ Xu et al.^30^ also reported increasing softening temperatures in their thermal analysis of 60P_2_O_5_–25 Bi_2_O_3_–(10 – *x*)CaO–5Sb_2_O_3_–*x*Gd_2_O_3_ (*x* = 0, 1, 2, 3, 4 mol %) glasses as well as decreased CTE values also interpreted in terms of the ionic field strength of Gd^3+^. The results reported for Eu_2_O_3_ replacing BaO in 50P_2_O_5_–(50 – *x*)BaO–*x*Eu_2_O_3_ (0 ≤ *x* ≤ 4 mol %) glasses are also consistent in this connection.^37^ It is then likely that the decrease in CTE indicating tighter networks in the current glasses reflects the incorporation of high-field strength Nd^3+^ (*F* = 3.105 Å^–2^) and Mn^2+^ (*F* = 2.903 Å^–2^) ions replacing Ba^2+^ ions with lower value (*F* = 0.922 Å^–2^).^27^ However, the effect of Mn^2+^ on the *T*_s_ appears more subtle as it was also observed with the *T*_g_ (*vide supra*). This is noticed with the *T*_s_ value of the 2Mn being somewhat lower than the undoped host despite the decreased CTE. Additional studies of glasses incorporating various manganese contents would then be desirable to help understand this effect.

### 3.3 Optical Properties

#### 3.3.1 Absorption Spectroscopy

**Figure 4:**
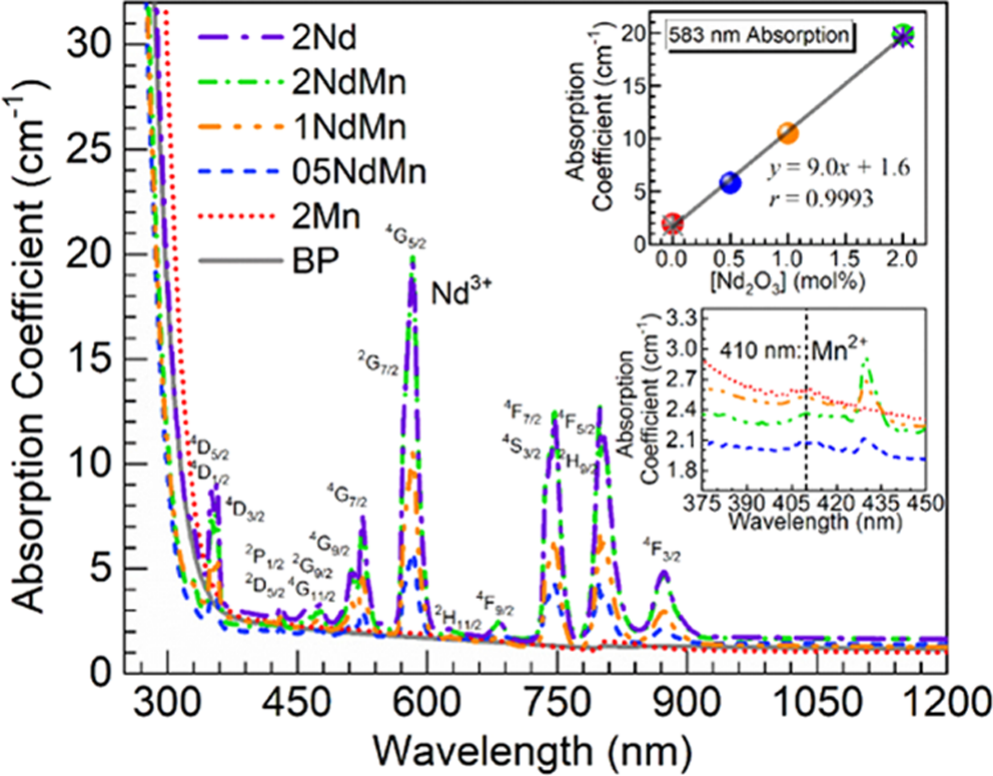
UV–vis–NIR absorption spectra obtained for the different glasses. The lower inset is an enlargement for the 2Mn, 05NdMn, 1NdMn, and 2NdMn glasses for appreciation of the Mn^2+^ absorption feature around 410 nm. The upper inset is a plot of the absorption intensity at 583 nm vs Nd_2_O_3_ concentration in the glasses (spheres—2Mn, 05NdMn, 1NdMn and 2NdMn; asterisks—BP and 2Nd references); the solid line is a linear fit to the data for the 2Mn, 05NdMn, 1NdMn, and 2NdMn glasses (equation and correlation coefficient, *r*, displayed).

Figure 4 shows the UV–vis–NIR optical absorption spectra obtained for the various glasses under consideration. The lower inset of Figure 4 is an enlargement for the 2Mn and 05-2NdMn glasses in the 375–450 nm range for better appreciation of the 410 nm absorption assigned to ^6^A_1_(S) → ^4^E(G),^4^A_1_(G) transitions in Mn^2+^ (d^5^) ions in octahedral coordination.^24,34,38,39^ The visible absorption of the 2Mn glass was otherwise resembling that of the undoped BP glass showing merely baseline absorption. This is consistent with the colorless appearance of the 2Mn glass and the lack of significant oxidation, which would otherwise produce a purple hue due to the absorption around 500 nm stemming from ^5^E_g_ → ^5^T_2g_ transition in Mn^3+^ (d^4^) ions in octahedral coordination.^35,38−41^ In addition, the 05-2NdMn glasses exhibit increasing intensities for the different Nd^3+^ transitions,^2,9,11^ spanning from the visible to the NIR. Further, the spectrum of the 2NdMn glass shows comparable intensity to the 2Nd reference concurring with a lack of interference from Mn^3+^. The absorption peak around 583 nm in connection with ^4^I_9/2_ → ^4^G_5/2_ + ^2^G_7/2_ transitions in Nd^3+^ ions is most prominently observed in the Nd-containing glasses. Hence, in the inset of Figure 4, the peak intensity is plotted as a function of the Nd_2_O_3_ concentration in the glasses (including the BP and 2Mn glasses with mere baseline absorption). The regression analysis performed on the data for the 2Mn and 05-2NdMn glasses yielded a slope of 9.0 cm^–1^/mol % with a correlation coefficient *r* of 0.9993, supporting a strong linear correlation. A similar outcome was found for the singly Nd-doped glasses with Nd_2_O_3_ replacing BaO up to 4 mol % in the 50P_2_O_5_–(50 – *x*)BaO–*x*Nd_2_O_3_ system, giving a slope at 8.9 cm^–1^/mol % and *r* at 0.9996.^27^ It is then herein similarly supported that the increasing Nd^3+^ amounts were successfully incorporated in the 05-2NdMn glasses.

**Figure 5:**
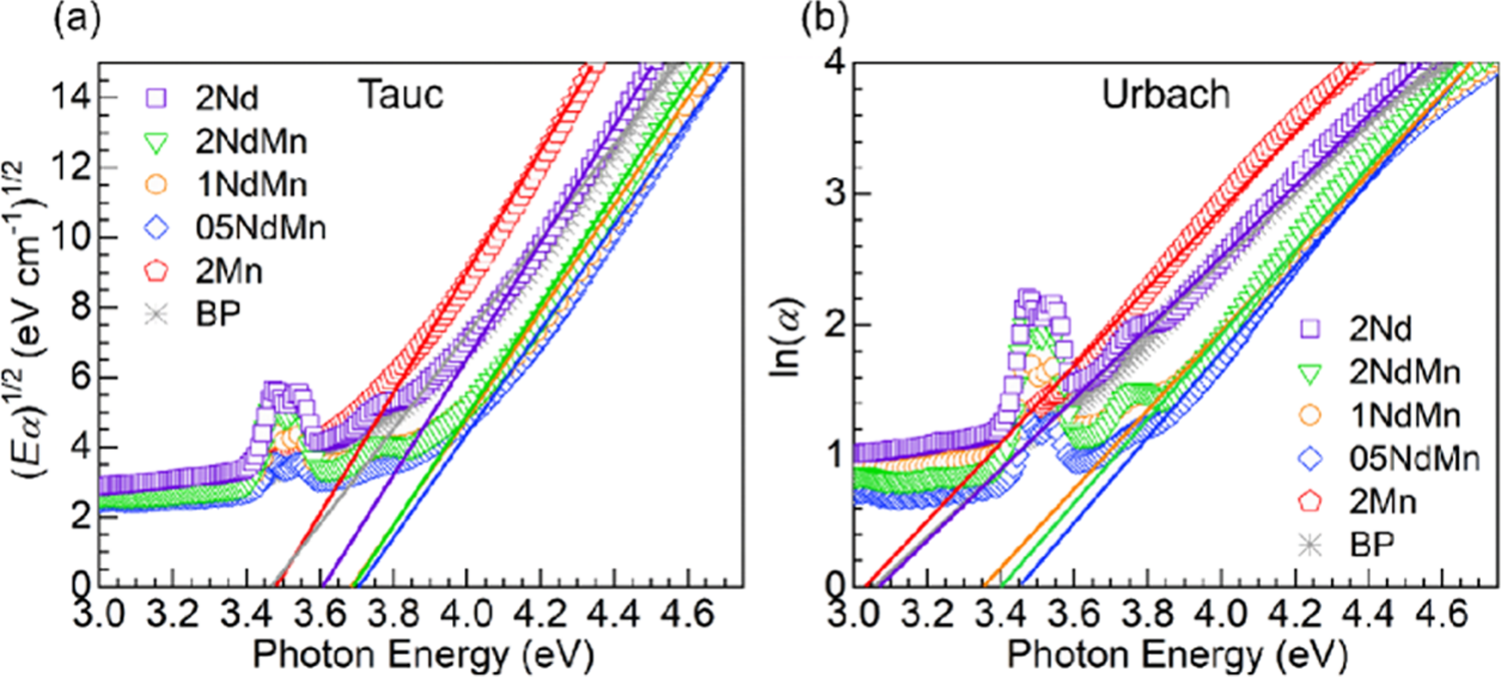
(a) Tauc plots (indirect band gaps) and (b) Urbach plots obtained for the various glasses. The solid traces represent the linear regressions to the data from where the optical band gaps (*E*_opt_) and Urbach energies (*E*_U_) were estimated (values shown in Table 5).

Some differences are also noticed in Figure 4, concerning the UV absorption edge of the glasses. This aspect may be further analyzed in the context of Tauc and Urbach plots.^5,11,41−43^ The following expression for the absorption coefficient, α, as a function of photon energy (*hν*), may be used to determine the optical band gap energies, *E*_opt_,
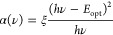
9where the exponent equal to 2 is linked to allowed-indirect transitions anticipated for glasses and ξ is a constant.^5,43^ The plots of (*Eα*)^1/2^ vs photon energy (*hν*) produced are presented in Figure 5a. These were employed to estimate *E*_opt_ from extrapolation of the linear portion to obtain the intercept on the energy axis.^11,27^ Then, the Urbach energy, *E*_U_, associated with band tailing reflecting defects/disorder may be assessed from the relation
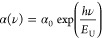
10where α_0_ is a constant and *hν* is the photon energy.^5,11,43^ Hence, the Urbach energy of the different glasses was evaluated from ln(α) vs *hν* plots, which are presented in Figure 5b. The linear regressions performed to the data in Figure 5a,b were such that the correlation coefficients (*r*) were between 0.997 and 0.998. The *E*_opt_ and *E*_U_ values deduced for all of the glasses are presented in Table 5 along with the errors derived from the fits. Compared to the BP and 2Mn glasses, the optical band gap energies appear higher for the Mn^2+^/Nd^3+^ codoped, although a clear trend with Nd^3+^ concentration is not established. Analogous results were reported for the singly Nd-doped glasses in the 50P_2_O_5_–(50 – *x*)BaO–*x*Nd_2_O_3_ (0 ≤ *x* ≤ 4 mol %) system that exhibited fluctuations.^27^ It was considered that the high-field strength Nd^3+^ ions could exert an influence by withdrawing electron density, thus lowering the top of the valence band, which can widen the band gap.^27^ Such an effect could ensue for the 05-2NdMn glasses studied here; however, an additional impact from Mn^2+^ is suggested by the higher band gap energies of this relative to the 2Nd reference with *E*_opt_ at 3.61 (±0.04) eV. With respect to the *E*_U_ values, it is observed in Table 5 that these fluctuate within the Mn^2+^ and Mn^2+^/Nd^3+^-containing glasses; however, lower energies are seen relative to the undoped host. This points to less structural disorder realized after replacing barium with manganese and neodymium. Furthermore, the 05-2NdMn-codoped glasses have lower Urbach energies than the 2Nd reference with an *E*_U_ of 0.368 (±0.004) eV, thus indicating a favorable effect from Mn^2+^ ions toward decreasing disorder. This opposes the reported effect of merely adding neodymium, which, at high concentrations, has been observed to promote higher structural disorder augmenting the localized states in the gap.^5,27^

**Table 5:** Optical Band Gaps (*E*_opt_) and Urbach Energies (*E*_U_) Estimated for the Various Glasses

glass	*E*_opt_ (eV)	*E*_U_ (eV)
BP	3.47 (±0.03)	0.380 (±0.003)
2Mn	3.49 (±0.06)	0.336 (±0.002)
05NdMn	3.71 (±0.05)	0.306 (±0.003)
1NdMn	3.69 (±0.05)	0.332 (±0.003)
2NdMn	3.69 (±0.05)	0.311 (±0.003)
2Nd	3.61 (±0.04)	0.368 (±0.004)

#### 3.3.2 PL Spectroscopy

**Figure 6:**
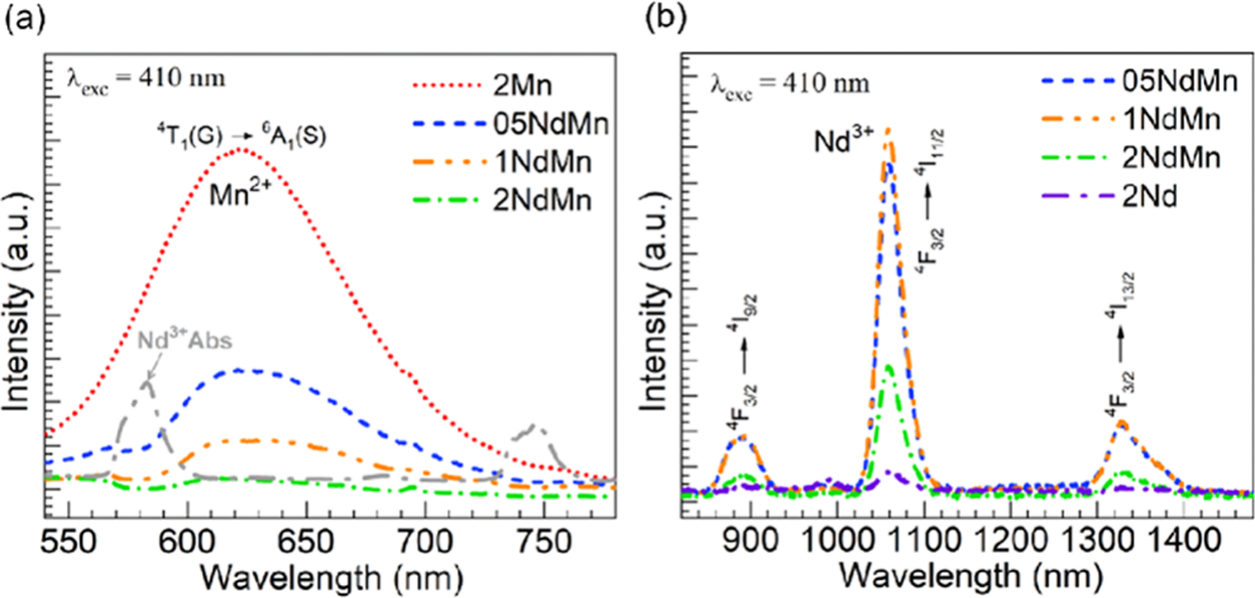
(a) Visible emission spectra obtained for the Mn-containing glasses under excitation at 410 nm; the absorption spectrum of the 2Nd glass is overlaid to show that Nd^3+^ absorption (Abs) coincides with the dips in emission. (b) NIR emission spectra obtained for the Nd-containing glasses under the 410 nm excitation.

**Figure 7:**
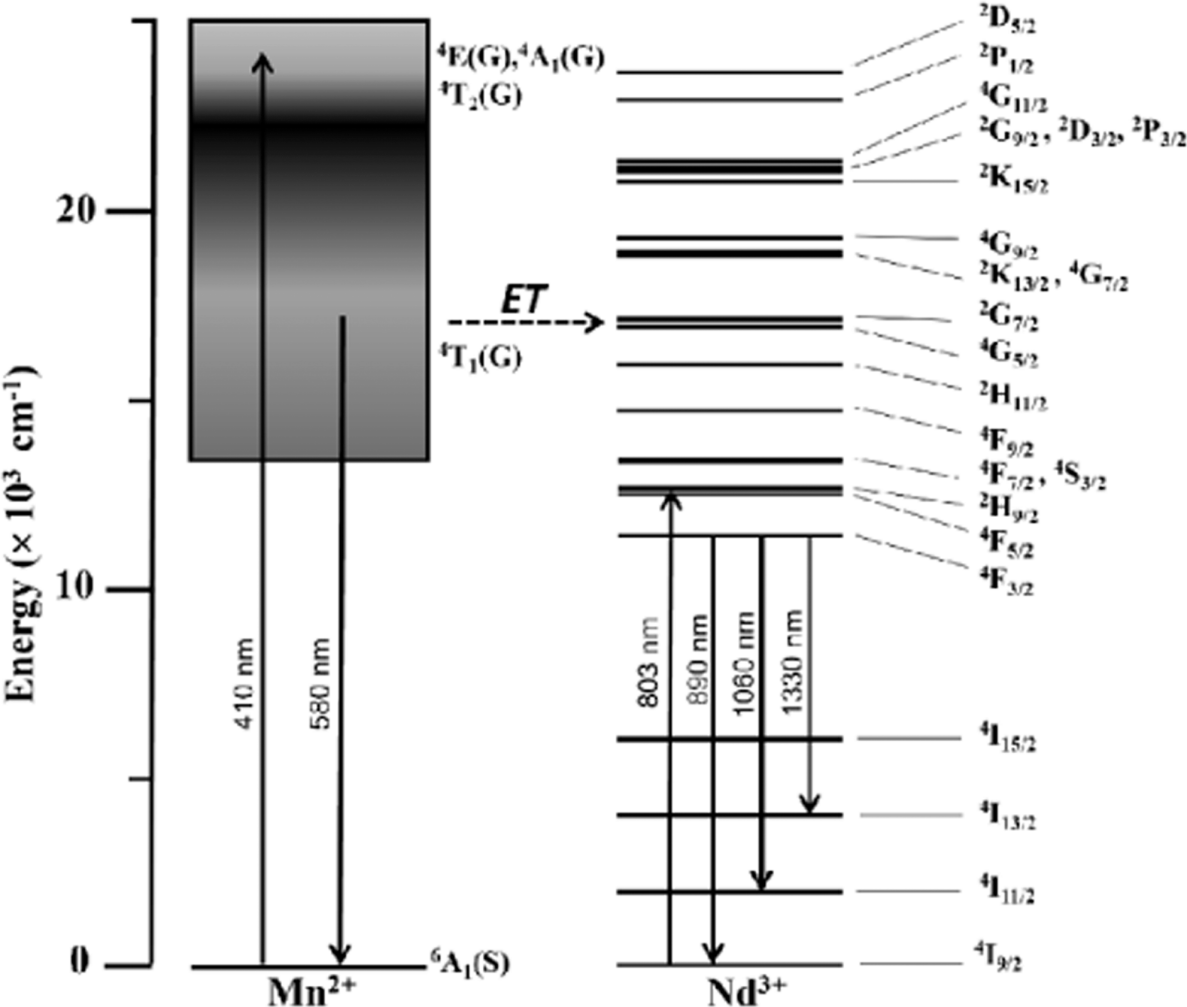
Simplified energy-level diagram of Mn^2+^ and Nd^3+^ ions illustrating the excitation of the ^6^A_1_(S) → ^4^E(G),^4^A_1_(G) transitions in Mn^2+^ at 410 nm, the emission from the ^4^T_1_(G) state at 580 nm, and the energy transfer (ET) to the ^4^G_5/2_ + ^2^G_7/2_ resonant levels in Nd^3+^ ions, leading to the radiative transitions from the ^4^F_3/2_ emitting state. The direct excitation of Nd^3+^ ions at 803 nm is also illustrated (nonradiative relaxations omitted).

Figure 6a,b shows PL spectra recorded under excitation of the ^6^A_1_(S) → ^4^E(G),^4^A_1_(G) transitions in Mn^2+^ at 410 nm seeking to assess the Mn^2+^ → Nd^3+^ energy transfer postulated.^15,16,23,24^ In Figure 6a, the emission is presented in the visible for the glasses that contain Mn^2+^ ions, namely the 2Mn and 05-2NdMn glasses. Then, in Figure 6b, the focus is on the NIR emission pertaining to Nd^3+^ ions, which are present in the 05-2NdMn and 2Nd glasses. The visible emission spectrum of the 2Mn glass in Figure 6a clearly shows the broad emission band with a maximum of around 620 nm, which is characteristic of the ^4^T_1_(G) → ^6^A_1_(S) transition in Mn^2+^ ions.^34,40,41^ The emission band becomes successively suppressed for the 05-2NdMn glasses even though the concentration of manganese in all of these was constant. A similar outcome was reported by Zhang et al.^24^ for Mn^2+^ emission in the Nd-doped 50P_2_O_5_–(50 – *x*)SrO–*x*MnO glasses, thus indicating the Mn^2+^ → Nd^3+^ energy transfer. Moreover, the spectra of the Mn^2+^/Nd^3+^ containing glasses show in Figure 6a a prominent dip around 580 nm. This coincides with the absorption of Nd^3+^ ions as perceived from the overlaid absorption spectrum of the 2Nd glass in Figure 6a. This latter trait distinguishes a resonant radiative Mn^2+^ → Nd^3+^ energy transfer while the uniform suppression in band emission is consistent with a nonradiative transfer.^24,44^ The simplified schematic shown in Figure 7 illustrates the excitation of Mn^2+^ ions at 410 nm along with the emission from the ^4^T_1_(G) state at 580 nm and the energy transfer to the ^4^G_5/2_ + ^2^G_7/2_ resonant levels in Nd^3+^ ions.

The nonradiative transfer from Mn^2+^ to Nd^3+^ has been previously studied by Ajithkumar and Unnikrishnan^23^ in phosphate glasses and is indicated to take place by the dipole–dipole mechanism. For this type of interaction, the transfer rate between a donor (*D*) and an acceptor (*A*), *k*_*DA*_, is given by
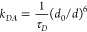
11where *d* is the *D–A* distance, *τ*_*D*_ is the donor lifetime in the absence of energy transfer, and *d*_0_ is the critical distance for which the transfer and spontaneous deactivation of the sensitizer have an equal probability.^23,44^ The critical *D–A* distance is given by
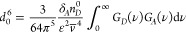
12where ν is the wavenumber, ε is the refractive index, *n*_*D*_^0^ is the donor quantum efficiency in the absence of energy transfer, ν̅ is the average wavenumber of the transition, δ_*A*_ is the integrated absorption cross section of the acceptor, and ∫_0_^∞^*G*_*D*_(ν)*G*_*A*_(ν) d*ν* represents the spectral overlap between donor emission and acceptor absorption. Hence, the energy transfer rate depends significantly on both the spectral overlap and the distance between the species interacting. Estimates for the critical distances reported by Ajithkumar and Unnikrishnan^23^ for the Mn^2+^–Nd^3+^*D–A* pair in phosphate glasses were in the range of 9.88–13.9 Å connected with energy transfer rates spanning from 6.25 to 633.2 s^–1^. The mean distances estimated herein between Mn^2+^ and Nd^3+^, *d*_Mn–Nd_, for the 05-2NdMn glasses are noticed in Table 2 to be in the 13.03–10.39 Å range. The decreasing *D–A* distances in the present glasses thus appear in good agreement with the expected for supporting an effective nonradiative transfer through the dipole–dipole mechanism.

The favorable interaction between Mn^2+^ ions as energy donors and Nd^3+^ ions as acceptors is further evidenced in the NIR emission spectra shown in Figure 6b. While the 2Nd glass without manganese exhibits rather weak emission, the 05-2NdMn glasses present clearly the typical Nd^3+^^4^F_3/2_ → ^4^I_9/2_, ^4^I_11/2_, ^4^I_13/2_ NIR transitions,^2,8,27^ which were observed around 890, 1060, and 1330 nm, respectively (illustrated in the schematic in Figure 7). Interestingly, the intensity of the ^4^F_3/2_ → ^4^I_11/2_ emission relevant for lasers was highest for the 1NdMn glass and then decreased drastically for the 2NdMn glass, which had the highest concentration of Nd^3+^ ions. This aspect points to the concentration quenching effect ensuing at high Nd^3+^ concentrations.^2,8,27^ Based on the results in Table 2, it is then seen that the most favorable Nd^3+^ concentration was then 2.993 × 10^20^ ions/cm^3^ with associated mean Nd^3+^–Nd^3+^ distances of 14.95 Å. Similar results were reported for the singly Nd-doped glasses in the 50P_2_O_5_–(50 – *x*)BaO–*x*Nd_2_O_3_ (0 ≤ *x* ≤ 4 mol %) system where the Nd^3+^ NIR emission achieved under 803 nm excitation first increased up to *x* = 1 and decreased thereafter.^27^ Sontakke et al.^2^ also observed for glasses with (100 – *x*)(20.95BaO–11.72Al_2_O_3_–56.12P_2_O_5_–6.79SiO_2_–3.91B_2_O_3_–0.51Nb_2_O_5_) + *x*Nd_2_O_3_ compositions that the maximum emission took place for 1 mol % Nd_2_O_3_ and decreased for higher concentrations. Nd^3+^ decay curves analysis for the 50P_2_O_5_–(50 – *x*)BaO–*x*Nd_2_O_3_ (0 ≤ *x* ≤ 4 mol %) system supported that the PL quenching was associated with the prevalence of the ^4^F_3/2_:^4^I_9/2_ → ^4^I_9/2_:^4^F_3/2_ excitation migration or “hopping” mechanism.^27^ Additional information from the Nd^3+^ emission decays will be likewise considered herein following Mn^2+^ decay kinetics analysis.

**Figure 8:**
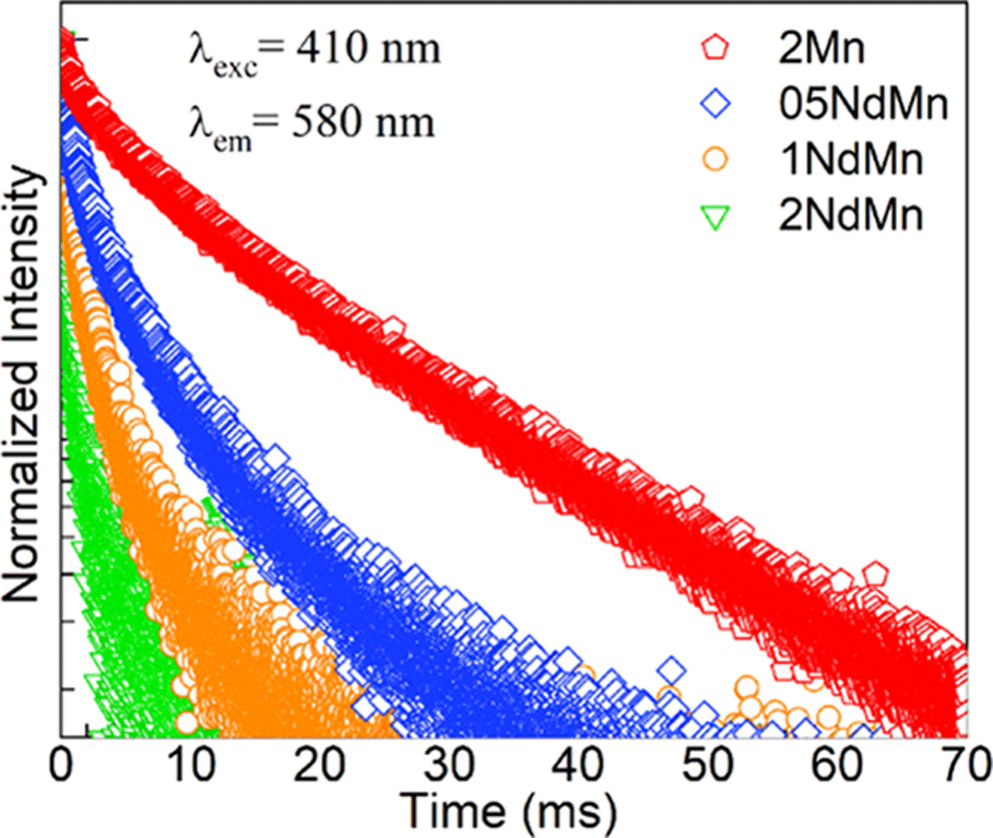
Semilog plots of the emission decay curves obtained for the Mn-containing glasses under excitation at 410 nm by monitoring emission at 580 nm.

Seeking insights into the energy transfer from Mn^2+^ to Nd^3+^ in the glasses, the decay dynamics of Mn^2+^ ions in the Mn-containing glasses are now evaluated. Figure 8 shows the decay curves obtained for the 2Mn and 05-2NdMn glasses exciting the ^6^A_1_(S) → ^4^E(G),^4^A_1_(G) transitions in Mn^2+^ at 410 nm. The emission was monitored at 580 nm for being resonant with the ^4^G_5/2_ + ^2^G_7/2_ levels in Nd^3+^ ions acting as acceptors (illustrated in Figure 7). The decays presented as normalized semilog plots exhibit complex behavior, where the increase in Nd^3+^ concentration clearly produces faster decays in the 05-2NdMn glasses. Zhang et al.^24^ observed a similar pattern for the Mn^2+^ emission decay curves obtained for the Nd-doped 50P_2_O_5_–(50 – *x*)SrO–*x*MnO glasses as a manifestation of the Mn^2+^ → Nd^3+^ energy transfer. The decay behavior of Mn^2+^ ions in glasses is certainly not single exponential in nature and is known to yield relatively slow decay times extending into the ms time scale.^24,38,41,45^ The decays were herein fit consistently with a biexponential function
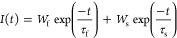
13where *I*(*t*) is the time-dependent luminescence intensity, *W*_f_ and *W*_s_ are pre-exponential weight factors, and τ_f_ and τ_s_ are fast and slow lifetimes of Mn^2+^ ions considered in the framework of interacting (e.g., Mn^2+^–Mn^2+^pairs) and isolated Mn^2+^ ions, respectively.^38,41^ The corresponding values estimated for the Mn-containing glasses are presented in Table 6 together with the errors stemming from the fits. Herein, an estimate of the population of Mn^2+^ ions with slow decay time, *P*_Mn,s_, can be obtained from the parameters in Table 6 by^46^
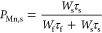
14The estimated percentages for the 2Mn, 05NdMn, 1NdMn, and 2NdMn glasses are 90.0, 80.3, 75.0, and 74.0%, respectively. Accordingly, the percentages of Mn^2+^ ions with fast decay time, *P*_Mn,f_, are 10.0, 19.7, 25.0, and 26.0%, for the 2Mn, 05NdMn, 1NdMn, and 2NdMn glasses, correspondingly. In the absence of neodymium, the percentage of Mn^2+^ ions with fast decay time (i.e., about 10% in the 2Mn glass) are considered to be interacting with other Mn^2+^ ions. However, an increment of the population of these with Nd^3+^ content points to their progressive involvement in the Mn^2+^ → Nd^3+^ energy transfer. Within the dipole–dipole interaction considered, the energy transfer efficiency, *η*, may be also estimated through the different decay times^47^ as
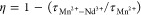
15where τ_Mn^2+^–Nd^3+^_ and τ_Mn^2+^_ are the lifetimes of the Mn^2+^ ions as donors in the presence and absence of the acceptor Nd^3+^ ions, respectively. Such efficiencies have been estimated in association with both fast and slow lifetime components in Mn^2+^ ions and are presented together with the lifetimes in Table 6. The lifetimes associated with isolated (τ_s_) and interacting (τ_f_) Mn^2+^ ions are both seen in Table 6 to decrease consistently for the 05-2NdMn glasses compared to the 2Mn reference, thus evidencing the nonradiative Mn^2+^ → Nd^3+^ transfer.^15,16,23,24^ Further, the transfer efficiencies in Table 6 increase with Nd_2_O_3_ contents in the 05-2NdMn glasses. This is consistent with the decrease in the mean distances (Table 2) estimated between Mn^2+^ and Nd^3+^ ions in these glasses within 13.03–10.39 Å. The efficiency values related to the fast decay component, *η*_f_, in Table 6 are nonetheless higher than the slow decay counterparts, with the most efficient transfer found for the 2NdMn glass with *η*_f_ = 91.8% efficiency. The data, therefore, suggests a greater susceptibility of the interacting Mn^2+^ ions such as pairs to participate in the Mn^2+^ → Nd^3+^ nonradiative transfer.

**Table 6:** Fast (τ_f_) and Slow (τ_s_) Lifetimes of Mn^2+^ Ions with Corresponding Weight Factors (*W*_f_, *W*_s_) Estimated from the Experimental Decays Obtained for the Mn-Containing Glasses under Excitation at 410 nm by Monitoring Emission at 580 nm^a^

glass	*W*_f_	*τ*_f_ (μs)	*η*_f_ (%)	*W*_s_	*τ*_s_ (μs)	*η*_s_ (%)
2Mn	0.324 (±0.001)	3686 (±8)		0.552 (±0.001)	19536 (±10)	
05NdMn	0.526 (±0.001)	1461 (±3)	60.4	0.297 (±0.001)	10518 (±14)	46.2
1NdMn	0.577 (±0.001)	741 (±2)	79.9	0.211 (±0.001)	6082 (±15)	68.9
2NdMn	0.668 (±0.003)	304 (±2)	91.8	0.168 (±0.002)	3433 (±33)	82.4

a The Calculated Mn^2+^ → Nd^3+^ Energy Transfer Efficiencies (η) Associated to Each Component Are Also Presented.

**Figure 9:**
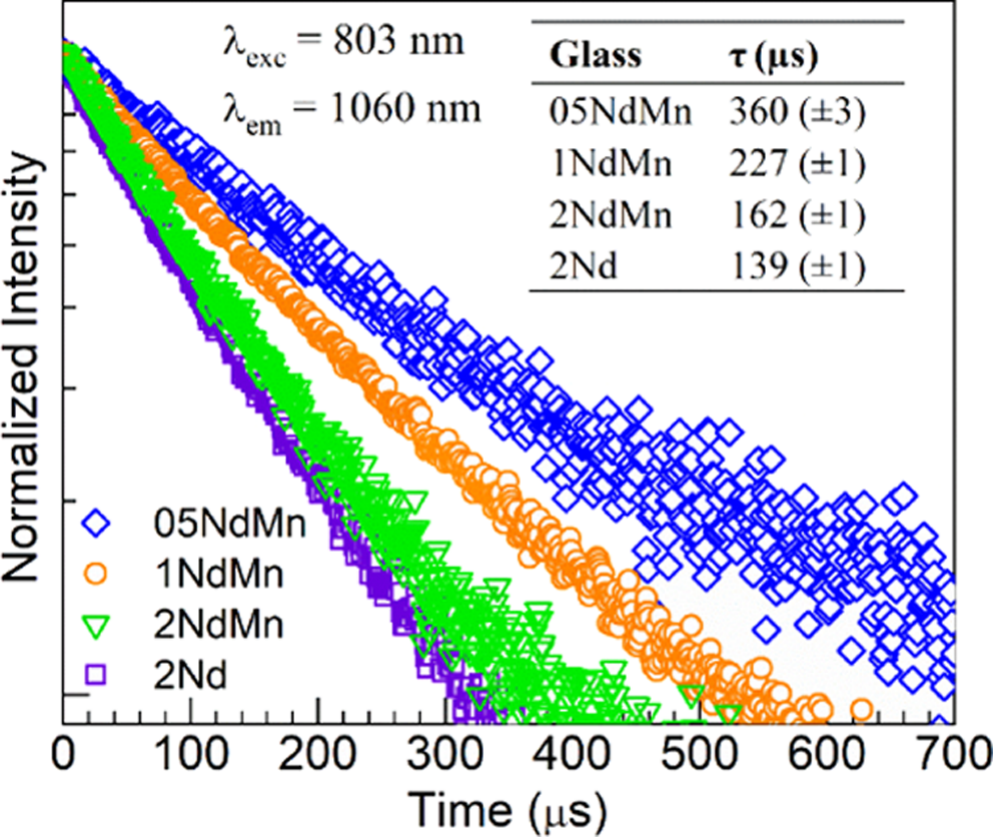
Semilog plots of emission decay curves obtained for the Nd-containing glasses under excitation at 803 nm with emission monitored at 1060 nm. The Table embedded as inset presents the ^4^F_3/2_ lifetimes (τ) estimated for Nd^3+^ ions in the glasses from single exponential fits to the data.

The data in Figure 8 and Table 6 indicated that the most favorable conditions for the Mn^2+^ → Nd^3+^ energy transfer were realized for the 2NdMn glass containing the highest concentration of Nd^3+^ ions. However, the emission spectra in Figure 6b showed that the best performance (e.g., for 1.06 μm lasing emission) was achieved for the 1NdMn glass. Hence, at this point, we turn our attention to the decay kinetics of Nd^3+^ ions for examining the basis for the PL evolution in Figure 6b. Figure 9 shows the Nd^3+^ emission decay curves obtained for the 05-2NdMn glasses and the 2Nd reference under excitation at 803 nm, accessing ^4^I_9/2_ → ^4^F_5/2_ + ^2^H_9/2_ transitions in Nd^3+^ ions while monitoring the ^4^F_3/2_ → ^4^I_11/2_ lasing emission at 1060 nm. The 803 nm excitation wavelength was chosen given its practical merit and to perform a comparison with reported Nd^3+^ lifetimes obtained similarly.^27^ The decay in Figure 9 appears to be faster with the increase in Nd^3+^ concentration in the glasses, wherein the behavior of the 2NdMn glass appears as the 2Nd reference prepared with the same amount of Nd_2_O_3_ at 2 mol %. The curves were fit in the context of first-order decay kinetics with a single-exponential function to obtain the different lifetimes (*τ*) for the ^4^F_3/2_ emitting state in Nd^3+^ ions.^27^ The lifetimes deduced alongside the estimated errors are shown in the Table embedded as inset in Figure 9. It is noticed that the 05NdMn glass has the longest ^4^F_3/2_ lifetime at 360 (±3) μs, and the lifetimes, thereafter, decreased to 227 (±1) and 162 (±1) μs for the 1NdMn and 2NdMn glasses, respectively. The overall shortening trend in the lifetimes indicates that significant interactions are taking place between Nd^3+^ ions,^2,8,27^ wherein the estimated mean distances, *d*_Nd–Nd_, in Table 2 decreased within the 18.80–11.89 Å range. The 2Nd reference glass then showed a similar lifetime to the 2NdMn glass, however with a slightly shorter value of 139 (±1) μs. The values obtained herein are in general comparable to the reported for Nd^3+^ in different phosphate glasses.^2,8,9,27^ Particularly similar are the Nd^3+^^4^F_3/2_ lifetimes reported for the 50P_2_O_5_–(50 – *x*)BaO–*x*Nd_2_O_3_ ternary system also studied under 803 nm excitation wherein decreasing lifetimes of 305 (±2), 212 (±1), and 143 (±1) μs were obtained for *x* = 0.5, 1, and 2 mol %, respectively.^27^ The PL intensity was, in such case, also highest for 1 mol % Nd_2_O_3_, and the analysis therein extended to 4 mol % Nd_2_O_3_ led to the conclusion that concentration quenching took place mainly via the ^4^F_3/2_:^4^I_9/2_ → ^4^I_9/2_:^4^F_3/2_ excitation migration mechanism.^27^ Although in the present work, the Nd_2_O_3_ concentration range was not high enough to attempt such analysis, and the results likewise suggest that excitation migration causes the PL quenching seen for the 2NdMn glass in Figure 6b. Analogously, the shorter lifetime obtained for the 1NdMn glass relative to the 05NdMn that showed a PL increase likely manifests the ^4^F_3/2_:^4^I_9/2_ → ^4^I_15/2_:^4^I_15/2_ cross relaxation pathway with less influence on emission output.^27^

**Figure 10:**
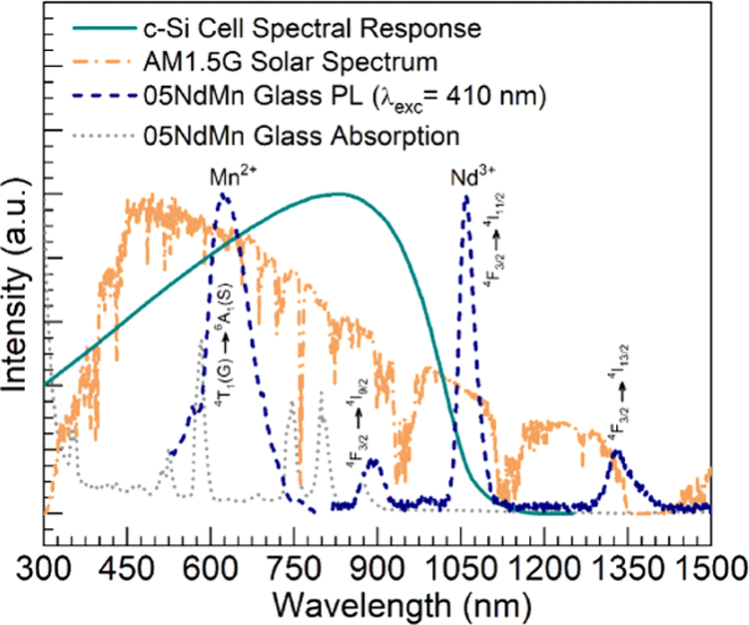
Overlay of absorption and PL spectra (λ_exc_ = 410 nm) of the 05NdMn glass with the spectral response of a c-Si cell and the AM1.5G solar spectrum (intensities were all normalized for comparison).

In Figure 10, the Mn^2+^/Nd^3+^ glass codoping strategy is put into perspective with the concept of solar spectral conversion. Here, the 05NdMn glass is chosen since it offers a suitable compromise between the suppressed red emission from the Mn^2+^ ions and the enhanced NIR emission from Nd^3+^ (Figure 6). Hence, the AM1.5G solar spectrum and the spectral response of a crystalline silicon (c-Si) cell^48^ are overlaid together with the absorption spectrum of the 05NdMn glass along with its PL spectra obtained under 410 nm excitation. It shows that various near-UV and visible absorption transitions in Mn^2+^ and Nd^3+^ ions overlap with the AM1.5G solar spectrum, which may provide for the necessary excitation. Then, the visible ^4^T_1_(G) → ^6^A_1_(S) emission from Mn^2+^ ions and the ^4^F_3/2_ → ^4^I_9/2_ NIR emission from Nd^3+^ ions are located in regions for which the c-Si cell has a suitable spectral response (also partially the ^4^F_3/2_ → ^4^I_11/2_ Nd^3+^ transition). The codoping of the glass with manganese(II) and neodymium(III) thus seems interesting for the development of a cover glass aiming to enhance the efficiency of solar cells.

## 4 Summary and Conclusions

Recapping, the melt-quenching technique was employed to prepare barium phosphate glasses containing red-emitting Mn^2+^ and NIR-emitting Nd^3+^ ions as interesting codopants for energy-relevant applications as solar spectral converters. The glasses were prepared by melting with 50P_2_O_5_–(48 – *x*)BaO–2MnO–*x*Nd_2_O_3_ with *x* = 0, 0.5, 1.0, and 2.0 mol % nominal compositions and studied by XRD, density, DSC, dilatometry, optical absorption, and PL spectroscopy measurements. The XRD evaluation supported the noncrystalline nature of the glasses, whereas the densities and molar volumes of the Mn^2+^/Nd^3+^ codoped glasses tended to increase with Nd_2_O_3_ content. The concentration of Mn^2+^ ions was similar for the codoped glasses within 3.010–2.971 × 10^20^ ions/cm^3^; however, Nd^3+^ concentration increased in the 1.505–5.943 × 10^20^ ions/cm^3^ range. The resulting Mn^2+^–Nd^3+^ interionic distances were then estimated to decrease within the 13.03–10.39 Å range. The thermal evaluation by DSC and dilatometry showed that the glass transition and softening temperatures of the codoped glasses increased with Nd_2_O_3_ content, indicating a stronger network was attained. Also evidenced was a shift in the onset of crystallization and the improvement in the thermal stability upon increasing Nd_2_O_3_ concentration. Further, the coefficient of thermal expansion decreased with Mn^2+^ and Nd^3+^, incorporation in the glasses pointing to increased glass rigidities likely connected with the high ionic field strength of Mn^2+^ and Nd^3+^ ions relative to Ba^2+^.

The UV–vis–NIR absorption evaluation of the codoped glasses supported the occurrence of Mn^2+^ ions absorbing around 410 nm due to ^6^A_1_(S) → ^4^E(G), ^4^A_1_(G) transitions, and the lack of Mn^3+^ ions, which otherwise would appear to absorb around 500 nm due to the spin-allowed ^5^E_g_ → ^5^T_2g_ transition. The evaluation also showed a linear increase in Nd^3+^ absorption (assessed at 583 nm in connection with ^4^I_9/2_ → ^4^G_5/2_ + ^2^G_7/2_ transitions) with Nd^3+^ ions concentration, which supported their effective incorporation in substitution of Ba^2+^. The analysis of glass absorption edges in the context of Tauc plots indicated in general higher band gap energies for the codoped glasses, likely related to the high-field strength of the codopants withdrawing electron density thus widening the energy gap. On the other hand, the Urbach energies tended to be lower, suggesting a tendency for decreased structural disorder. The PL spectroscopy inquiry showed the consistent suppression of the ^4^T_1_(G) → ^6^A_1_(S) transition red emission band around 620 nm from Mn^2+^ ions with increasing Nd^3+^ concentration. Also exhibited was a prominent dip around 580 nm in correspondence with the absorption of Nd^3+^ ions via ^4^I_9/2_ → ^4^G_5/2_ + ^2^G_7/2_ transitions. The Mn^2+^ emission decay curves analyzed in the context of interacting and isolated Mn^2+^ ions revealed shorter lifetimes in connection with increased Mn^2+^ → Nd^3+^ transfer efficiencies. Herein, the interacting Mn^2+^ ions showed a higher predisposition to participate in the resonant nonradiative transfer, exhibiting the highest efficiency at 91.8% for Nd_2_O_3_ content of 2.0 mol %. Nevertheless, exciting Mn^2+^ ions at 410 nm led to the sensitized Nd^3+^ NIR emission with the highest intensity exhibited for 1.0 mol % Nd_2_O_3_. Emission decay analysis of the ^4^F_3/2_ emitting state in Nd^3+^ ions showed lifetime shortening throughout; the PL quenching of the NIR emission was then considered to follow most likely the ^4^F_3/2_:^4^I_9/2_ → ^4^I_9/2_:^4^F_3/2_ excitation migration or “hoping” mechanism. Finally, considering the compromise between the red and NIR emissions obtained for the Mn-containing glass with 0.5 mol % Nd_2_O_3_, its spectral properties were put into context with the concept of solar spectral conversion. The overlap between glass absorption and emission with the AM1.5G solar spectrum and spectral response of a c-Si cell, respectively, suggests a potential of the codoping strategy for the energy-related application.

## Data Availability

The data underlying this study are available in the published article.
